# Deciphering the Complex Distribution of Human Immunodeficiency Virus Type 1 Subtypes among Different Cohorts in Northern Tanzania

**DOI:** 10.1371/journal.pone.0081848

**Published:** 2013-12-11

**Authors:** Harr F. Njai, Fiona M. Ewings, Eric Lyimo, Vincent Foulongne, Dhamira Ngerageza, Aika Mongi, Deogratius Ssemwanga, Aura Andreasen, Balthazar Nyombi, Tony Ao, Denna Michael, Mark Urassa, Jim Todd, Basia Zaba, John Changalucha, Richard Hayes, Saidi H. Kapiga

**Affiliations:** 1 Mwanza Intervention Trials Unit, National Institute for Medical Research, Mwanza, Tanzania; 2 London School of Hygiene and Tropical Medicine, London, United Kingdom; 3 MRC Tropical Epidemiology Group, London School of Hygiene and Tropical Medicine, London, United Kingdom; 4 National Institute for Medical Research, Mwanza, Tanzania; 5 Laboratoire de Virologie, University of Montpellier, Montpellier, France; 6 MRC/UVRI Uganda Research Unit on AIDS, Entebbe, Uganda; 7 Kilimanjaro Christian Medical Centre, Moshi, Tanzania; Fudan University, China

## Abstract

**Background:**

Increased understanding of the genetic diversity of HIV-1 is challenging but important in the development of an effective vaccine. We aimed to describe the distribution of HIV-1 subtypes in northern Tanzania among women enrolled in studies preparing for HIV-1 prevention trials (hospitality facility-worker cohorts), and among men and women in an open cohort demographic surveillance system (Kisesa cohort).

**Methods:**

The polymerase encompassing partial reverse transcriptase was sequenced and phylogenetic analysis performed and subtype determined. Questionnaires documented demographic data. We examined factors associated with subtype using multinomial logistic regression, adjusted for study, age, and sex.

**Results:**

Among 140 individuals (125 women and 15 men), subtype A1 predominated (54, 39%), followed by C (46, 33%), D (25, 18%) and unique recombinant forms (URFs) (15, 11%). There was weak evidence to suggest different subtype frequencies by study (for example, 18% URFs in the Kisesa cohort versus 5–9% in the hospitality facility-worker cohorts; adjusted relative-risk ratio (aRR) = 2.35 [95% CI 0.59,9.32]; global p = 0.09). Compared to men, women were less likely to have subtype D versus A (aRR = 0.12 [95% CI 0.02,0.76]; global p = 0.05). There was a trend to suggest lower relative risk of subtype D compared to A with older age (aRR = 0.44 [95% CI 0.23,0.85] per 10 years; global p = 0.05).

**Conclusions:**

We observed multiple subtypes, confirming the complex genetic diversity of HIV-1 strains circulating in northern Tanzania, and found some differences between cohorts and by age and sex. This has important implications for vaccine design and development, providing opportunity to determine vaccine efficacy in diverse HIV-1 strains.

## Introduction

Human immunodeficiency virus type 1 (HIV-1) is characterised by extensive genetic variability, as a consequence of high replication and mutation rates and frequent recombination [1], [2]. Based on phylogenetic analysis, HIV-1 strains have been divided into four major phylogenetic groups: M, N, O and P. Group M, the predominant circulating group responsible for the global HIV-1 pandemic [3], is divided into nine subtypes (designated A-D, F-H, and J-K) [2]. Some of the viral strains, such as subtypes A and F, have been further sub-divided into sub-subtypes A1-A5, and F1–F2, respectively [4]. In addition, different subtypes may recombine to form circulating recombinant forms (CRFs), which continue to be transmitted from one individual to another, or unique recombinant forms (URFs) if there is no evidence of transmission from the patient in which they arose to another. Currently, at least 58 CRFs and numerous URFs have been identified [5].

Most known HIV-1 groups and subtypes have been reported in Africa; this region shows the greatest diversity of circulating HIV-1 viral strains [4]. Tanzania is among the countries in Africa severely affected by the HIV epidemic, with an estimated prevalence of HIV among the general adult population of 5.7% in 2007/2008 [6]. The distribution of HIV-1 subtypes in different regions of Tanzania gives an impression of a genetically more complex and diverse epidemic than some of its neighbouring countries. During the initial phase of the epidemic in Tanzania, HIV-1 subtypes A and D predominated and were found in similar proportions [7]. Subtype C was subsequently introduced during the late 1980s-early 1990s from neighbouring southern African countries [4], [7]. Currently, subtypes A1 and C are the main circulating strains among the general population [8]–[10] and in high-risk groups [9], [11]. This is in contrast to the epidemics in other East African countries, which consist of mainly subtypes A1 and D [12]–[14]. Tanzania also reports the highest prevalence of recombinant forms within the East African region [8], [9].

Increased understanding of the genetic diversity of HIV-1 is challenging but important in the development of an effective vaccine [15], and may impact transmission, diagnosis, disease progression, viral burden, response to treatment and emergence of ART resistance [1], [16]. Limited information is available on the distribution of HIV-1 subtypes in Tanzania and no studies have been conducted in Mwanza region of northern Tanzania. We describe the distribution of HIV-1 subtypes and associated risk factors in this region, where we developed local laboratory capacity to conduct HIV-1 viral genotyping. We included a general population cohort and two high-risk cohorts to compare the distribution of HIV-1 subtypes among different populations and geographical locations.

## Methods

### Ethics statement

The studies were approved by the Ethics Committees of the Tanzania National Institute for Medical Research (NIMR), Kilimanjaro Christian Medical Centre (KCMC) and London School of Hygiene and Tropical Medicine. All participants involved in this study gave written informed consent (all aged ≥18 years). Study documents were stored in a secure location to ensure participants' confidentiality. Staff were trained on confidentiality issues, research ethics and protection of human subjects. Those testing HIV-positive were referred to care and treatment centres.

### Study populations

**Figure 1:**
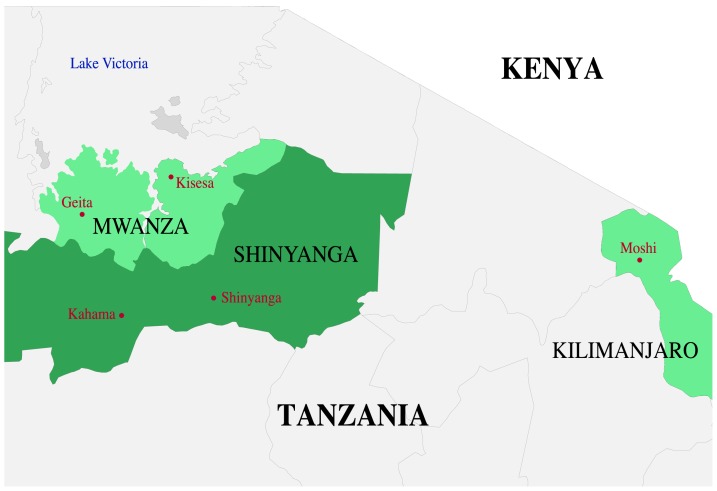
Map of the study areas in northern Tanzania. Created using StatSilk (2013; StatPlanet: Interactive Data Visualization and Mapping Software. http://www.statsilk.com).

We analysed data from women participating in two prospective cohort studies conducted in preparation for trials of candidate microbicides and HIV-1 vaccines (hospitality facility-worker cohorts), and among men and women from the general population enrolled in the Kisesa open cohort for demographic health surveillance (Kisesa cohort; Figure 1). We aimed to enrol 50 individuals from each of the three cohorts, and analysed one sample per participant.

### Study procedures for hospitality facility-worker cohorts

Participants in the cohorts were recruited between 2008–2010 from hotels, restaurants, bars, guesthouses, food-sellers at makeshift facilities, and shops selling traditionally-brewed beer in the towns of Geita, Kahama, and Shinyanga near Mwanza city (microbicides-preparedness study) and Moshi town (vaccines-preparedness study). During a screening visit, women underwent a brief interview to collect limited information about demographic characteristics, behavioural risk factors and other information to determine their study eligibility, and a blood sample was collected for HIV testing. Women were eligible if they were aged 18–44 years, willing to undergo HIV testing and receive results, and not planning to move away from the recruitment site for the duration of the study.

Women fulfilling the inclusion criteria were invited to come to an enrolment visit within 4 weeks. HIV-negative women were eligible for enrolment in both the microbicides-preparedness and the vaccines-preparedness studies. In the vaccines-preparedness study, HIV-positive women with CD4 cell count ≥350 cells/mm^3^ and who were ART-naïve with no indication to start ART were also enrolled. At enrolment, structured face-to-face interviews were conducted to obtain information about socio-demographic characteristics, employment, reproductive history, sexual behaviour and work mobility. All women enrolled in the study were scheduled to return to the clinic after every three months for 12 months. During each visit, similar interviews, clinical examinations and blood and genital sample collection were performed as at enrolment. Blood and genital samples were transported to either a laboratory at NIMR in Mwanza city (microbicides-preparedness cohort) or KCMC in Moshi (vaccines-preparedness cohort) for further processing.

For this virological study, we aimed to recruit all HIV seroconverters identified during the follow-up of the cohorts. In order to reach the target sample size, we also aimed to recruit all HIV-positive women enrolled in the vaccines-preparedness study, and a random sample of HIV-positive women identified during the screening process in the microbicides-preparedness study.

### Study procedures for Kisesa cohort

The Kisesa cohort has been described previously [17] and will be reviewed here briefly. Kisesa is located about 20 kms from Mwanza city, along the main road connecting Mwanza with major cities in Kenya. Demographic surveillance has been conducted at approximately half-yearly intervals since 1994. In addition, detailed sero-surveys are conducted approximately every three years to collect blood for HIV-1 testing and sexual behaviour data. Typically around 70% of all adults aged ≥15 years who were invited participated in the sero-surveys [18]. A sero-survey was conducted in 2010–2011 (sero 6), in which dry blood spots were taken from 9,276 participants and tested for HIV in the NIMR laboratory in Mwanza. Individuals who requested voluntary counselling and testing (VCT) in order to know their HIV status were given a separate rapid test by trained VCT counsellors, and a plasma blood sample was taken for storage in NIMR laboratories. For the purpose of this study, we randomly selected plasma samples from individuals who were HIV-1 positive during the sero 6 sero-survey.

### HIV-1 diagnosis

Testing for infections was performed according to standard operating procedures in each of the NIMR and KCMC laboratories. In the hospitality facility-worker cohorts, HIV rapid testing was performed at screening in parallel using SD Bioline HIV-1/2 3.0 (Standard Diagnostics, Inc., Korea) and Determine HIV-1/2 (Alere Medical, Co., Ltd, Japan) tests. If the rapid tests were positive or discordant, HIV infection was confirmed in the respective laboratories using either third generation Murex HIV 1.2.O (Abbott UK, Dartford, Kent, England) and Vironostika HIV Uniform II plus O (bioMérieux Bv, The Netherlands) enzyme-linked immunosorbent assays (ELISAs; at NIMR laboratory for the microbicides-preparedness study), or only Vironostika HIV Uniform II plus O ELISA (at KCMC laboratory for the vaccines-preparedness study). In the microbicides-preparedness study, samples discrepant or indeterminate on ELISA were tested for P24 Antigen (Genetics Systems HIV-1 Ag EIA, Bio-rad Laboratories, Marnes-La Coquette, France) and if positive were classified as HIV-positive. Samples negative for P24 antigen were tested by Western Blot (INNO-LIA, HIV I/II score, Innogenetics NV, Gent, Belgium). At enrolment and follow-up visits, HIV testing was done using ELISAs as per the screening algorithm. In the vaccines-preparedness study, the HIV testing algorithm used at screening was applied at enrolment and throughout the follow-up period.

In the Kisesa cohort, HIV tests were run on the DBS samples using two ELISAs in series (Vironostika HIV-Uniform II Plus O (bioMérieux Bv, the Netherlands) and if positive then Enzygnost Anti-HIV 1/2 Plus (Dade Behring Neward, DE, USA)); only those with two positive results were considered HIV-positive.

### Sample collection, viral load measurement and genotyping

Among those selected for this virological study, whole blood was collected to isolate peripheral blood mononuclear cells (PBMC) and aliquot plasma. Frozen plasma samples were shipped to University of Montpellier (UoM), France, for viral load (VL) determination and HIV-1 genotyping. Viral RNA was extracted using the MagNa pure Compact Nucleic Acid Isolation Kit (Roche Diagnostics). HIV VL was determined at UoM by RT-PCR (Generic HIV viral Load, Biocentric) and at KCMC Biotechnology Laboratory, Moshi, Tanzania by RT-PCR (Abbott M2000) for 10 women who seroconverted after the shipment of samples to UoM.

The reverse transcriptase (RT) gene encompassing part of the polymerase (HXB2 positions 2530–3334) was amplified by nested PCR using primers RT19: (5′-GGA-CAT-AAA-GCT-ATA-GGT-ACA-G-3′) and RT20: (5′-CTG-CCA-GTT-CTA-GCT-CTG-CTT-C-3′) or MJ3: (5′-AGT-AGG-ACC-TAC-ACC-TGT-CA-3′) and MJ4: (5′-CTG-TTA-GTG-CTT-TGG-TTC-CTC-T-3′) in the first round and A1: (5′-TTG-GTT-GCA-CTT-TAA-ATT-TTC-CCA-TTA-GTC-CTA-TT-3′) and NE1: (5′-CCT-ACT-AAC-TTC-TGT-ATG-TCA-TTG-ACA-GTC-CAG-CT-3′) in the second-round reactions [19], [20]. The PCR products were purified with a QIAquick PCR purification kit (Qiagen, Valencia, CA) and sequenced using the BigDye® Terminator v3.1 Cycle Sequencing Kits (Applied Biosystems, Foster City, CA) on an 8-cappillary ABI Prism 3500 Genetic Analyzer (Applied Biosystems). The sequencing primers were A2 (2545→2564): 5′-ATT-TTC-CCA-TTA-GTC-CTA-TT-3′ and NE2 (3300←3319): (5′- ATG-TCA-TTG-ACA-GTC-CAG-CT-3′) [19], [20]. Sequencing products were purified using Centri-Sep™ spin columns (Princeton Separations, Inc New Jersey, USA).

### Genotyping assay quality control

To successfully implement the genotyping assay developed by UoM at NIMR, we randomly selected from our archive 39 duplicate plasma samples, which were previously processed in UoM. Genotyping was performed independently at NIMR and data generated compared to data obtained from UoM. Sequences were assembled and edited using Sequencher, version 4.10.1 (GeneCodes Corporation, Ann Arbor, MI) and aligned using ClustalW in Bio-Edit Sequence Alignment Editor v7.0.9.0 [21], yielding a percentage identity for NIMR versus UoM results. We determined the proportion achieving at least 98% [22] identity, and the proportion with matching subtypes.

### Genetic analysis of cohort samples

The nucleotide sequence chromatogram files were assembled and edited using Sequencher version 4.10.1 (GeneCodes Corporation, Ann Arbor, MI). Sequences were aligned with reference pure subtype sequences of HIV-1 group M downloaded from Los Alamos National library (LANL) HIV Sequence Database [5] using ClustalW in Bio-Edit Sequence Alignment Editor v7.0.9.0 [21]. The REGA HIV-1 sub-typing tool [23] at the Stanford database was used to assign subtypes [24]. Phylogenetic trees were inferred in SeaView version 4.3.5 [25], using HKY85 model [26] and Neighbor-joining method [27] with 1000 bootstrap replicate values [28].

### Statistical methods

Participant characteristics were summarised by cohort and the following variables: HIV-1 seroconverter (defined as ≤36 months between the last negative and first positive test dates), sex, age, education, main job, ethnicity, religion, age at first sex, number of partners in lifetime, and last 12 months and HIV VL. We investigated the characteristics associated with HIV-1 subtype using multinomial logistic regression (with outcome of subtype categories), adjusted for study, age and sex. The variables were added to the model one at a time, and we included those with overall p<0.10 in a multivariable model, from which we removed those no longer meeting the p<0.10 threshold. Finally, we considered adding each of the omitted variables (retaining those with p<0.10). Continuous variables were categorised or their effects were assumed linear (see Table 1). Analyses were performed using Stata (StataCorp. 2009. *Stata Statistical Software: Release 11*. College Station, TX: StataCorp LP).

**Table 1:** Participant characteristics by cohort.

	Cohort			Overall	P-value (Χ^2^ test)
	Female hospitality facility-workers from 3 towns near Mwanza city	Female hospitality facility-workers in Moshi town	Kisesa open cohort study		
N	53 (100%)	42 (100%)	45 (100%)	140 (100%)	
HIV INFECTION					
HIV status [1]					0.03
Prevalent	39 (74%)	29 (69%)	41 (91%)	109 (78%)	
Seroconverter	14 (26%)	13 (31%)	4 (9%)	31 (22%)	
DEMOGRAPHICS					
Sex					[2]
Male	0 (0%)	0 (0%)	15 (33%)	15 (11%)	
Female	53 (100%)	42 (100%)	30 (67%)	125 (89%)	
Age, years	30 [25,36]	30 [26,37]	35 [27,43]	31 [26,38]	
<25	13 (25%)	6 (14%)	5 (11%)	24 (17%)	0.2
25–29	12 (23%)	12 (29%)	9 (20%)	33 (24%)	
30–34	14 (26%)	12 (29%)	8 (18%)	34 (24%)	
35–39	5 (9%)	7 (17%)	9 (20%)	21 (15%)	
≥40	9 (17%)	5 (12%)	14 (31%)	28 (20%)	
Education					0.03
≤Incomplete primary	15 (28%)	6 (14%)	20 (44%)	41 (29%)	
Complete primary	33 (62%)	33 (79%)	24 (53%)	90 (64%)	
≥Secondary	5 (9%)	3 (7%)	1 (2%)	9 (6%)	
Main job					<0.001
Missing	0	0	1	1	
Waitress	22 (42%)	22 (52%)	0 (0%)	44 (32%)	
Other hospitality facility staff [3]	31 (58%)	20 (48%)	1 (2%)	52 (37%)	
Farming	0 (0%)	0 (0%)	27 (61%)	27 (19%)	
Other	0 (0%)	0 (0%)	11 (25%)	11 (8%)	
No work	0 (0%)	0 (0%)	5 (11%)	5 (4%)	
Ethnicity					<0.001
Sukuma	25 (47%)	1 (2%)	40 (89%)	66 (47%)	
Chaga	8 (15%)	18 (43%)	0 (0%)	26 (19%)	
Other	20 (38%)	23 (55%)	5 (11%)	48 (34%)	
Religion					0.03
Missing	1	0	0	1	
Christian	40 (77%)	33 (79%)	36 (80%)	109 (78%)	
Muslim	12 (23%)	9 (21%)	5 (11%)	26 (19%)	
Other	0 (0%)	0 (0%)	4 (9%)	4 (3%)	
Marital status					<0.001
Missing	2	0	0	2	
Married/cohabiting	13 (25%)	9 (21%)	32 (71%)	54 (39%)	
Widowed/separated/ divorced	31 (61%)	22 (52%)	11 (24%)	64 (46%)	
Single	7 (14%)	11 (26%)	2 (4%)	20 (14%)	
SEXUAL BEHAVIOUR					
Age at first sex, years					0.63
Missing	7	1	6	14	
<16	13 (28%)	8 (20%)	10 (26%)	31 (25%)	
≥16	33 (72%)	33 (80%)	29 (75%)	95 (75%)	
Number of lifetime sexual partners	9	8	12	29	0.07
Missing	20 (45%)	22 (65%)	25 (76%)	67 (60%)	
0–4	16 (36%)	10 (29%)	6 (18%)	32 (29%)	
5–9	8 (18%)	2 (6%)	2 (6%)	12 (11%)	
≥10					
Number of sexual partners in last 12 months					<0.001
Missing	5	2	0	7	
0–1	20 (42%)	28 (70%)	39 (87%)	87 (65%)	
2–10	28 (58%)	12 (30%)	6 (13%)	46 (35%)	
LABORATORY RESULTS					
HIV VL [4]					0.08
Undetectable	3 (6%)	0 (0%)	0 (0%)	3 (2%)	
Detectable	50 (94%)	42 (100%)	45 (100%)	137 (98%)	
HIV VL, log_10_ copies/ml [5]	4.3 [3.5,4.8]	4.1 [3.4,4.7]	5.2 [4.1,5.7]	4.5 [3.6,5.2]	0.001 [6]

%) for categorical variables and median [interquartile range] for continuous variables. Percentages are of non-missing values. Results are n (

[1] HIV seroconverters defined as those with ≤36 months between last negative and first positive test dates.

[2] P-value omitted since differences are by design.

[3]Including food preparation, *mamalishe* and bar work.

[4] Lower limit of detection was 300 copies/ml, except for the 10 subsequent seroconverters, where the lower limit of detection was 75 copies/ml.

[5] Imputed as half the lower limit of detection, for those with undetectable HIV VL.

[6] Comparing categories split by the overall median.

### Sequence Data

Sequences from this study were deposited in GenBank under the following accession numbers: KC831606–KC831741.

## Results

### Participant characteristics

We selected 162 participant samples for analysis of which 11 (7%) were PCR-negative, 10 (6%) failed sequencing or had poor sequences, and 1 (1%) was dropped due to sample mix-up in the quality control check, leaving 140 (86%) samples that were included in the present study. Of these, 53 (38%), 42 (30%) and 45 (32%) were from the microbicides-preparedness study, the vaccines-preparedness study, and the Kisesa cohort, respectively.

In Table 1, we present the characteristics by study cohort among participants included in the final genotyping analysis. Overall, 31 (22%) were identified as HIV-1 seroconverters, the majority of whom by design were female hospitality facility-workers, since Kisesa surveys only occur every 3 years. Among seroconverters, the median time from the first positive test date to blood collection was 2 (interquartile range [IQR]: 0, 9) months. The majority of participants were female (89%). Female hospitality facility-workers were younger than participants from the Kisesa cohort (median 30 versus 35 years). Women from the hospitality facility-worker cohorts predominantly worked as waitresses or in similar roles (42% in the towns near Mwanza city and 52% in Moshi town), compared to mainly farming in the Kisesa cohort (61%). Hospitality facility-workers were less likely to be married or cohabiting (25% in the towns near Mwanza city and 21% in Moshi town), compared to the Kisesa cohort (71%).

Overall, 93% of participants had not progressed to secondary level education, 47% were of Sukuma ethnicity and 78% were Christian. Participants from the hospitality facilities in Moshi and the Kisesa cohort had lower numbers of sexual partners over their lifetime and in the last 12 months, compared to hospitality facility-workers in towns near Mwanza city (p = 0.07 and <0.001, respectively). The median HIV VL was 4.3, 4.1 and 5.2 log_10_ copies/ml in the microbicides-preparedness study, vaccines-preparedness study and Kisesa cohort, respectively.

### HIV genotyping

#### Quality Control

From the list of samples already processed in UoM, we randomly selected 39 for HIV genotyping at NIMR. Of these, four samples were not sequenced at UoM (2 failed PCR, 2 failed sequencing) and one did not have a matching sample in the NIMR archive. Of the remaining 34 samples, 32 samples were successfully sequenced and analysed, with 27 (84%) samples meeting the ≥98% identity threshold. We failed to determine the subtype of one sequence generated at UoM due poor quality of sequence. Of the remaining 31 sequences, 28 (90%) had concordant HIV subtypes. The three discrepant sequences were of subtypes D versus C/D, C versus C/H, and CRF10_CD versus C, for NIMR versus UoM, respectively.

#### HIV-1 subtype classification and distribution

**Figure 2:**
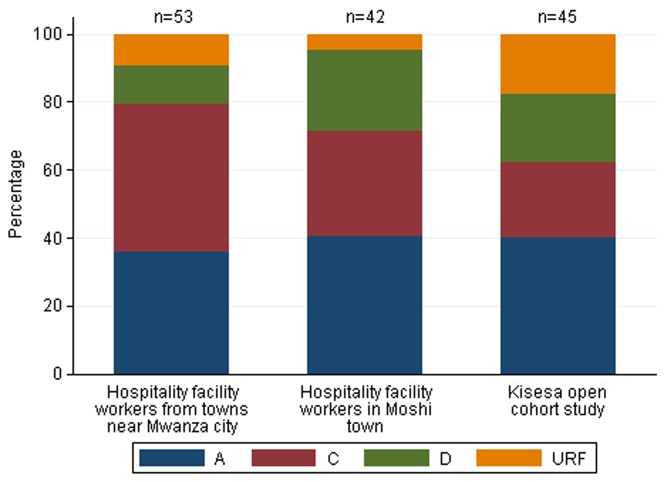
Distribution of HIV-1 subtype by cohort. Χ^2^ test, p = 0.16.

**Figure 3:**
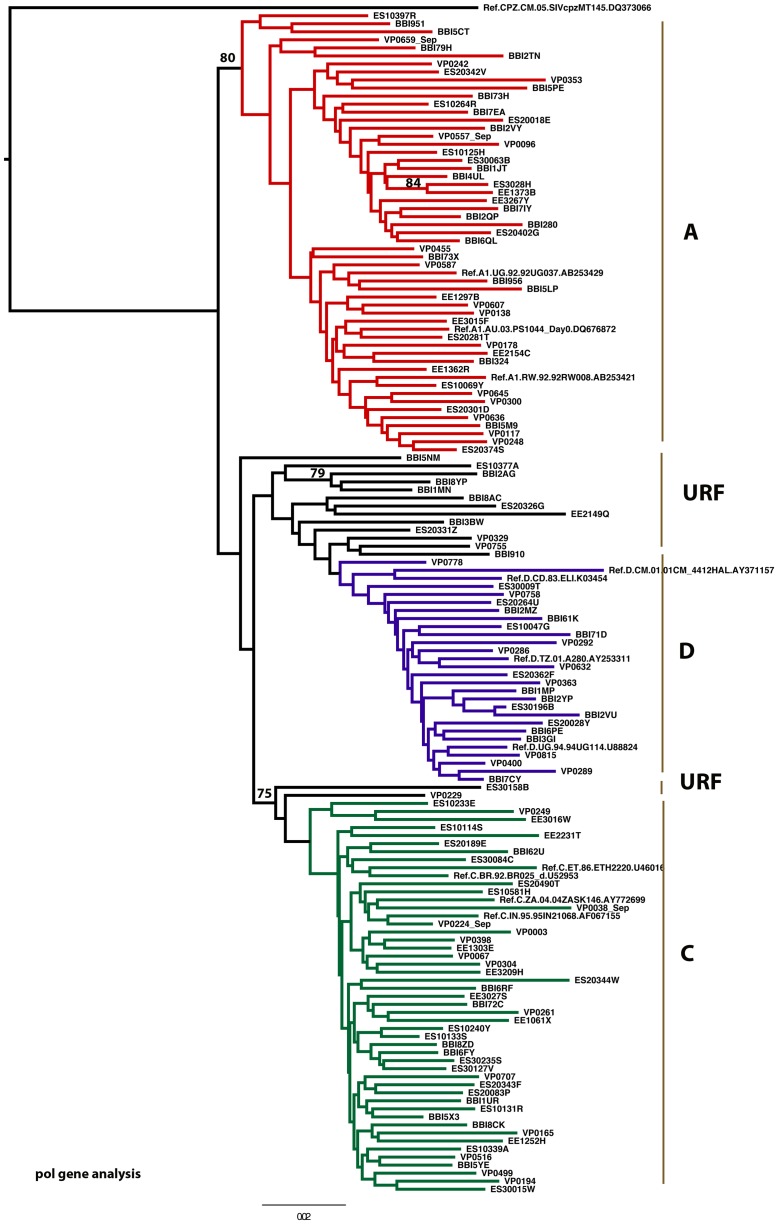
pol gene analysis tree. Results are presented for the 136 sequences which passed for phylogenetic analysis.

We successfully generated sequences from 140 participants, out of which 126 (90%) samples were sequenced at UoM and the remaining 14 (10%) samples were sequenced at NIMR where we developed local laboratory capacity to conduct genotyping. The latter samples were from participants who seroconverted after shipment of samples to UoM or had sample mix up at UoM necessitating retesting of samples at NIMR. Of 140 participants, 54 (39%) infections were subtype A, 46 (33%) subtype C, 25 (18%) subtype D and 15 (11%) URFs (Figure 2). The latter included subtypes C/D (n = 5), B/D (n = 2), D/B (n = 2), A/C (n = 1), A/D (n = 1), C/A (n = 1), C/H (n = 1), D/A (n = 1) and D/C (n = 1). After exclusion of 4 short sequences, 136 were passed for phylogenetic analysis (Figure 3).

In Table 2, we show the distribution of HIV-1 subtypes by participant characteristics. The proportions of participants infected with subtype A virus were similar across the three study populations, while the proportion with subtype C was relatively higher among hospitality facility-workers in towns near Mwanza city (43%) compared to the other populations (31% among hospitality facility-workers in Moshi town and 22% in the Kisesa cohort). Participants in the Kisesa cohort were more likely to be infected with URF viruses (18%) compared to hospitality facility-workers in towns near Mwanza city (9%) and Moshi town (5%). The proportion of participants infected with subtype D was lowest among hospitality facility-workers in towns near Mwanza (11%).

**Table 2:** Subtypes by participant characteristics.

	Subtype				Overall	P [1]
	A	C	D	URF		
N	54 (39%)	46 (33%)	25 (18%)	15 (11%)	140 (100%)	
Study						0.16
Hospitality facility-workers from towns near Mwanza city	19 (36%)	23 (43%)	6 (11%)	5 (9%)	53 (100%)	
Hospitality facility-workers in Moshi town	17 (40%)	13 (31%)	10 (24%)	2 (5%)	42 (100%)	
Kisesa open cohort study	18 (40%)	10 (22%)	9 (20%)	8 (18%)	45 (100%)	
HIV INFECTION						
HIV status [2]						0.43
Prevalent	43 (39%)	33 (30%)	22 (20%)	11 (10%)	109 (100%)	
Seroconverter	11 (35%)	13 (42%)	3 (10%)	4 (13%)	31 (100%)	
DEMOGRAPHICS						
Sex						0.12
Male	5 (33%)	3 (20%)	6 (40%)	1 (7%)	15 (100%)	
Female	49 (39%)	43 (34%)	19 (15%)	14 (11%)	125 (100%)	
Age, years	32 [27,40]	32 [26,39]	29 [26,33]	33 [25,36]	31 [26,38]	
<25	8 (33%)	9 (38%)	5 (21%)	2 (8%)	24 (100%)	0.27
25–29	13 (39%)	7 (21%)	9 (27%)	4 (12%)	33 (100%)	
30–34	11 (32%)	14 (41%)	6 (18%)	3 (9%)	34 (100%)	
35–39	7 (33%)	5 (24%)	5 (24%)	4 (19%)	21 (100%)	
≥40	15 (54%)	11 (39%)	0 (0%)	2 (7%)	28 (100%)	
Education						0.14
≤Incomplete primary	20 (49%)	8 (20%)	7 (17%)	6 (15%)	41 (100%)	
Complete primary	33 (37%)	32 (36%)	17 (19%)	8 (9%)	90 (100%)	
≥Secondary	1 (11%)	6 (67%)	1 (11%)	1 (11%)	9 (100%)	
Main job						0.18
Missing	0	0	0	1	1	
Waitress	18 (41%)	13 (30%)	10 (23%)	3 (7%)	44 (100%)	
Other hospitality facility staff [3]	18 (35%)	24 (46%)	6 (12%)	4 (8%)	52 (100%)	
Farming	12 (44%)	5 (19%)	7 (26%)	3 (11%)	27 (100%)	
Other/no work	6 (38%)	4 (25%)	2 (13%)	4 (25%)	16 (100%)	
Ethnicity						0.88
Sukuma	26 (39%)	23 (35%)	9 (14%)	8 (12%)	66 (100%)	
Chaga	11 (42%)	7 (27%)	5 (19%)	3 (12%)	26 (100%)	
Other	17 (35%)	16 (33%)	11 (23%)	4 (8%)	48 (100%)	
Religion						0.06
Missing	0	0	1	0	1	
Christian	37 (34%)	39 (36%)	20 (18%)	13 (12%)	109 (100%)	
Muslim	16 (62%)	7 (27%)	2 (8%)	1 (4%)	26 (100%)	
Other	1 (25%)	0 (0%)	2 (50%)	1 (25%)	4 (100%)	
Marital status						0.78
Missing	0	2	0	0	2	
Married/cohabiting	18 (33%)	19 (35%)	9 (17%)	8 (15%)	54 (100%)	
Widowed/separated/ divorced	28 (44%)	20 (31%)	11 (17%)	5 (8%)	64 (100%)	
Single	8 (40%)	5 (25%)	5 (25%)	2 (10%)	20 (100%)	
SEXUAL BEHAVIOUR						
Age at first sex, years						0.67
Missing	7	5	1	1	14	
<16	14 (45%)	8 (26%)	5 (16%)	4 (13%)	31 (100%)	
≥16	33 (35%)	33 (35%)	19 (20%)	10 (11%)	95 (100%)	
Number of lifetime sexual partners						0.41
Missing	10	14	3	2	29	
0–4	24 (36%)	19 (28%)	13 (19%)	11 (16%)	67 (100%)	
5–9	13 (41%)	10 (31%)	8 (25%)	1 (3%)	32 (100%)	
≥10	7 (58%)	3 (25%)	1 (8%)	1 (8%)	12 (100%)	
Number of sexual partners in last 12 months						0.76
Missing	1	5	1	0	7	
0–1	37 (43%)	27 (31%)	14 (16%)	9 (10%)	87 (100%)	
2–10	16 (35%)	14 (30%)	10 (22%)	6 (13%)	46 (100%)	
LABORATORY RESULTS						
HIV VL, log_10_ copies/ml [4]	4.3 [3.5,4.9]	4.6 [3.4,5.3]	4.5 [3.7,5.4]	5.0 [3.8,5.6]	4.5 [3.6,5.2]	
<Median	29 (41%)	22 (31%)	14 (20%)	6 (8%)	71 (100%)	0.73
≥Median	25 (36%)	24 (35%)	11 (16%)	13 (9%)	69 (100%)	

Results are n (%) for categorical variables and median [interquartile range] for continuous variables. Percentages are of non-missing values. URF = unique recombinant form.

[1] P-value from Χ^2^ test.

[2] HIV seroconverters defined as those with ≤36 months between known last negative and first positive test dates.

[3] Including food preparation, *mamalishe* and bar work.

[4] Imputed as half the lower limit of detection (300 copies/ml, except for the 10 subsequent seroconverters, where the lower limit of detection was 75 copies/ml), for those with undetectable HIV VL. Median is as shown in Table 1 (4.5 log_10_ copies/ml).

The distribution of HIV-1 subtypes by demographic and behavioural factors was not uniform. The crude data suggest that participants who were aged ≥40, those who had incomplete primary versus higher education, Muslims compared to other religions, and those reporting ≥10 lifetime sexual partners had relatively higher proportions of subtype A. We also examined a number of factors which were collected among female hospitality facility-workers only (data not shown), including duration of working in the facilities, number of places lived in the past year, number of times in the past year spent >1 week away from home, problematic alcohol drinking and past year history of exchanging money or gifts for sex. There was no evidence that the HIV-1 subtype distribution differed by these factors.

Our final regression model included study population, age and sex only, since none of the other covariates reached p<0.10 after adjustment for these factors. As shown in Table 3, there was some evidence to suggest independent associations with subtype by study population, age and sex (p = 0.09, 0.05, and 0.05, respectively), although the confidence intervals were generally wide. In particular, comparing females versus males, there was a lower relative risk of being infected with subtype D compared to subtype A (adjusted relative-risk ratio (aRR) = 0.12, 95% CI 0.02, 0.76). Furthermore, there were trends to suggest lower relative risk of subtypes C, D and URF compared to subtype A with older age (aRR = 0.86, 95% CI 0.54,1.38; aRR = 0.44, 95% CI 0.23,0.85; and aRR = 0.67, 95% CI 0.34,1.33, per 10 years, respectively).

**Table 3:** Study-, age- and sex-adjusted associations between subtype and participant characteristics.

	Study-, age- and sex-adjusted relative risk ratio (95% CI), relative to subtype A			P [1]
	Subtype C	Subtype D	URF	
Study				0.09
Hospitality facility-workers from towns near Mwanza city	1 (reference)	1 (reference)	1 (reference)	
Hospitality facility-workers in Moshi town	0.63 (0.25,1.62)	2.00 (0.58,6.83)	0.46 (0.08,2.67)	
Kisesa open cohort study	0.47 (0.15,1.44)	0.92 (0.19,4.53)	2.35 (0.59,9.32)	
HIV status [2]				0.26
Prevalent	1 (reference)	1 (reference)	1 (reference)	
Seroconverter	1.38 (0.52,3.65)	0.39 (0.09,1.67)	1.81 (0.43,7.56)	
Sex				0.05
Male	1 (reference)	1 (reference)	1 (reference)	
Female	0.79 (0.14,4.55)	0.12 (0.02,0.76)	2.00 (0.18,21.9)	
Age, per 10 years	0.86 (0.54,1.38)	0.44 (0.23,0.85)	0.67 (0.34,1.33)	0.05
Education				0.18
≤primary	1 (reference)	1 (reference)	1 (reference)	
≥secondary	7.09 (0.79,63.9)	1.57 (0.09,27.9)	3.30 (0.17,62.7)	
Job				0.57
Waitress/recreational-related [3]	1 (reference)	1 (reference)	1 (reference)	
Farmer	0.90 (0.14,5.91)	1.41 (0.13,15.7)	0.40 (0.03,4.91)	
Other	2.03 (0.73,5.65)	1.02 (0.24,4.34)	1.29 (0.22,7.61)	
Ethnicity				0.22
Sukuma	1 (reference)	1 (reference)	1 (reference)	
Non-Sukuma	0.68 (0.23,2.04)	3.03 (0.59,15.5)	2.20 (0.45,10.7)	
Religion				0.21
Christian	1 (reference)	1 (reference)	1 (reference)	
Non-Christian	0.38 (0.14,1.05)	0.45 (0.12,1.68)	0.40 (0.08,2.02)	
Marital status				0.61
Married	1 (reference)	1 (reference)	1 (reference)	
Widowed/separated/divorced	0.48 (0.18,1.29)	1.33 (0.35,5.08)	0.49 (0.12,2.02)	
Single	0.36 (0.09,1.54)	0.89 (0.17,4.69)	0.67 (0.09,5.04)	
Age at first sex, years				0.7
<16	1 (reference)	1 (reference)	1 (reference)	
≥16	1.81 (0.66,4.99)	1.51 (0.44,5.21)	1.23 (0.32,4.81)	
Number of life partners [4]				0.22
0–4	1 (reference)	1 (reference)	1 (reference)	
5–9	0.72 (0.24,2.11)	0.93 (0.28,3.07)	0.15 (0.02,1.34)	
≥10	0.31 (0.06,1.55)	0.16 (0.01,1.69)	0.21 (0.02,2.31)	
Number of partners in last 12 months				0.59
0–1	1 (reference)	1 (reference)	1 (reference)	
2–10	0.88 (0.33,2.35)	1.47 (0.45,4.77)	2.31 (0.51,10.4)	
HIV VL, per log_10_ copies/ml [5]	1.29 (0.85,1.96)	1.16 (0.68,1.97)	1.35 (0.74,2.47)	0.59

[1] P-value from likelihood ratio test, relative to model with study, age and sex only.

[2] HIV seroconverters defined as those with ≤36 months between last negative and first positive test dates.

[3] Including food preparation, *mamalishe* and bar work.

[4] Only 33% of men reported the number of lifetime partners, so it was not possible to fit a model with both sex and number of lifetime partners, therefore the results reported are study- and age-adjusted only (and compared to model with study and age only).

[5] Imputed as half the lower limit of detection (300 copies/ml, except the 10 subsequent seroconverters, where the lower limit of detection was 75 copies/ml), for those with undetectable HIV VL.

## Discussion

This study describes the molecular epidemiology of HIV-1 among men and women in a peri-urban general population and among women known to be at increased risk of HIV-1 infection in northern Tanzania. We observed multiple HIV-1 subtypes in the study populations, confirming the complex genetic diversity of HIV-1 strains circulating in these areas.

Our results show that HIV-1 subtypes A (39%) and C (33%) were the most prevalent, followed by subtype D (18%) and URF (11%). This is consistent with previous studies conducted in northern Tanzania [8], [11], [29]. To our knowledge, this is the first study to investigate the molecular epidemiology of HIV-1 in the towns near Mwanza city, the main Tanzanian urban centre on the shores of Lake Victoria. To our knowledge, only one study has previously described the distribution of HIV-1 subtypes around Lake Victoria, and this was conducted in Bukoba town of Kagera region [8]. Findings from the previous and present studies suggest that HIV-1 subtypes A and C may be the most prevalent subtypes in these regions around Lake Victoria.

We observed a substantial proportion of recombinant viruses across the three cohorts, with the general population cohort in Kisesa having the highest frequency. However, this was a relatively lower frequency than previously reported [7]. This confirms that re-infection (or superinfection) may occur among people infected with HIV, leading to recombinants that are subsequently transmitted [30]. The high genetic variability of the virus may pose significant problems in the specificity and/or sensitivity of serological and molecular diagnostic tests [31]. In Uganda, genetic variability was shown to have an effect on response to ART [32] and the development of HIV dementia among individuals with advanced immunosuppression [32] and this has implications for the treatment of HIV infection. Likewise, an effective anti-HIV-1 vaccine should elicit efficient cellular as well as humoral immune responses and, in particular, broadly neutralising antibodies able to target the largest number of HIV-1 genetic forms [1], [15], [16], [33].

We found only weak evidence of a difference in subtype distribution by cohort, with a suggestion of higher prevalence of subtype C, and lower subtype D, in hospitality facility-workers from towns near Mwanza city compared to hospitality facility-workers in Moshi town and the Kisesa general population. Higher prevalence of subtype C among hospitality facility-workers may be because they are more likely to be exposed to sex partners from areas where subtype C is highly prevalent. We observed that females were less likely to be infected with subtype D, compared to subtype A. Furthermore, we found trends towards higher relative prevalence of subtype A with older age. This may be due to subtype A, along with subtype D, being more predominant at earlier stages of the epidemic, with subtype C increasing over time, or may be related to better prognosis among those infected with subtype A compared to subtype D [34], [35]. While this is a small study, these differences warrant further investigation.

In the present study, we genotyped samples from multiple cohorts recruited in five towns in northern Tanzania, including women known to be at increased risk of infection and men and women from the general population. Thus, we were able to compare our results across the study populations with different socio-demographic and risk behaviour profiles. These strengths should be considered in light of a number of limitations. Firstly, many of the participants were enrolled as prevalent cases, and so the time of infection was unknown and may have been many years earlier. This is relevant both because subtype distributions may change over time, and because data on some risk factors, particularly sexual behaviour, may apply to periods after infection. Secondly, we amplified only a partial region of polymerase gene and this may lead to misclassification of HIV-1 recombinant forms. However, sequencing this gene provides opportunity for future studies to examine primary HIV drug resistance mutations [36]. Thirdly, the sample size was small, and included only a relatively small number of men and seroconverters; the latter constrained our ability to examine trends in the distribution over time.

In summary, we have described the molecular epidemiology of HIV-1 in northern Tanzania across divergent populations, and confirmed the complex genetic diversity of HIV-1 strains circulating in the study areas. These results underscore the need for appropriate HIV-1 vaccine development to address the multifaceted HIV epidemic. As reported in previous studies, HIV-1 subtypes A and C were the most prevalent viral strains. We also observed a substantial proportion of recombinant viruses, although somewhat lower than previously reported. We found some differences in the distribution of HIV-1 subtypes between cohorts, and by age and sex. This study provides the groundwork for future HIV-1 vaccine research to be conducted in northern Tanzania. The populations involved in this study provide opportunity to assess the efficacy of candidate vaccines against diverse HIV-1 strains.
